# Genome Sequence of *Porphyromonas gingivalis* Strain A7A1-28

**DOI:** 10.1128/genomeA.00021-17

**Published:** 2017-03-09

**Authors:** Gary Xie, Ryan P. Chastain-Gross, Myriam Bélanger, Dibyendu Kumar, Joan A. Whitlock, Li Liu, William G. Farmerie, Collin L. Zeng, Hajnalka E. Daligault, Cliff S. Han, Thomas S. Brettin, Ann Progulske-Fox

**Affiliations:** a Bioenergy and Biome Sciences (B-11), Bioscience Division, Los Alamos National Laboratory, Los Alamos, New Mexico, USA; b Department of Oral Biology, University of Florida, Gainesville, Florida, USA; c Center for Molecular Microbiology, University of Florida, Gainesville, Florida, USA; d Interdisciplinary Center for Biotechnology Research, University of Florida, Gainesville, Florida, USA

## Abstract

*Porphyromonas gingivalis* is an oral opportunistic pathogen. Sequenced *P. gingivalis* laboratory strains display limited diversity in antigens that modulate host responses. Here, we present the genome sequence of A7A1-28, a strain possessing atypical fimbrillin and capsule types, with a single contig of 2,249,024 bp and a G+C content of 48.58%.

## GENOME ANNOUNCEMENT

*Porphyromonas gingivalis* is an anaerobic bacterium (1) associated with periodontal disease (2–4) and multiple systemic diseases (5–7). *P. gingivalis* may manipulate host responses to orchestrate dysbiosis and disease (8, 9), potentially through extensive variation in fimbrillin genotypes (10–12) and capsule serotypes (13, 14). Notably, common *P. gingivalis* laboratory strains display limited fimbrillin and capsule diversity (11, 15–18), limiting laboratory modeling of periodontal disease. This has extended to genome sequencing: fimbrillin type I, capsule absent (15, 17, 19) (ATCC 33277 [20] and 381 [21]), and fimbrillin type IV, capsule K1 (15, 17, 22, 23) (W83 [24] and A7436 [25]). Fimbrillin and capsule information is unavailable for other sequenced laboratory strains of *P. gingivalis* (26–28). A7A1-28 is a widely available strain that exhibits fimbrillin type II and capsule K3 (15, 17, 19). Isolated in 1985 by Neiders and Chen at SUNY-Buffalo (Buffalo, NY, USA) from a type 2 diabetes patient (29, 30), A7A1-28 stimulates *in vitro* responses that dramatically differ from those elicited by W83 or ATCC 33277 (13, 14); however, these differences have not been evaluated at the genome level. This study was undertaken to determine the complete genome sequence of A7A1-28 and facilitate investigations of the variety of host responses elicited by strains of *P. gingivalis*.

*P. gingivalis* strain A7A1-28 was obtained from Kesavalu Lakshmyya (University of Florida) and grown as previously described (31). Genomic DNA was obtained using the Wizard gDNA purification kit (Promega) and processed to generate shotgun and 8-kb paired-end libraries, which were sequenced using the 454 Life Sciences GS-20 instrument (32) (Roche). A total of 468,259 reads of 235,999,749 bp, with an average read length of 504 bp, were generated.

The GS-20 reads were assembled using Velvet version 0.7.63 (https://www.ebi.ac.uk/~zerbino/velvet) (33) and Newbler version 2.3 (Roche) (32). Gaps between contigs were closed by editing in Consed (http://www.phrap.org/consed/consed.html) (34–36) and by PCR-augmented Sanger sequencing. The genome was annotated using the RAST (http://metagenomics.anl.gov) (37) and IMG-ER servers (http://img.jgi.doe.gov/er) (38) and then amended using Gene Prediction Improvement Pipeline software (39).

The genome of *P. gingivalis* A7A1-28 has approximately 94-fold coverage and contains a single contig of 2,249,024 bp (G+C content of 48.58%). A total of 1,982 genes were annotated, which included 1,915 predicted coding sequences (CDSs), 53 tRNAs, 12 rRNAs, and one tmRNA. There are 229 subsystems in the genome. Further, 191 protein metabolism, 128 cofactors, vitamins, prosthetic groups, and pigments, 65 RNA metabolism, 94 DNA metabolism, 99 carbohydrate, and 17 membrane transport subsystem features were observed.

The annotated *P. gingivalis* A7A1-28 genome was compared to *P. gingivalis* strains W83, ATCC 33277, and TDC60 using RAST (37) and IMG-ER (38). All-to-all BLASTP comparisons of predicted protein sequences showed that A7A1-28 possesses 119 strain-specific CDSs, of which 98 are annotated as hypothetical proteins. Further, A7A1-28 contains a variety of mobile genetic elements, including seven *Bacteroides* conjugative transposons absent from W83, ATCC 33277, and TDC60. Genome synteny analysis revealed that the gene order in A7A1-28 resembles that of A7436 and AJW4, suggesting that local mutations may generate unique phenotypes observed in A7A1-28.

The availability of the A7A1-28 genome aids investigators in efforts to decipher interactions between *P. gingivalis* and host tissue, which are critical to homeostasis in the subgingival microbiome.

### Accession number(s).

This genome sequencing project was deposited in GenBank under the accession number CP013131. The version described is the first version.
